# A new perspective on Wendan decoction: attenuation of CUMS-induced anxiety in mice by regulating gut microbiota and neuroinflammation

**DOI:** 10.3389/fmicb.2025.1708868

**Published:** 2025-11-21

**Authors:** Zixuan Guo, Qianqian Wang, Yunge Cao, Baiyan Wang, Boyi Zhang, Jiawei Huang, Yuanyuan Niu, Junhong Huang, Zilong Wang, Shuying Feng

**Affiliations:** 1 Medical College, Henan University of Chinese Medicine, Zhengzhou, China; 2 Henan Engineering Research Center for Chinese Medicine Foods for Special Medical Purpose, Zhengzhou, China

**Keywords:** Wendan decoction, anxiety, gut microbiota, neuroinflammation, chronic unpredictable mild stress

## Abstract

**Purpose:**

To study the effects of Wendan decoction (WDD) on anxiety in mice that have been exposed chronic unpredictable mild stress (CUMS) and to look into the underlying mechanisms from the perspective of regulating neuroinflammation and gut microbiota.

**Methods:**

The CUMS program was administered to C57BL/6 J mice to mimic chronic exposure to persistent and uncontrollable stresses. Alterations in anxiety-like behaviors were documented via behavioral tests. After euthanasia, pro-inflammatory cytokines in colonic and hippocampal tissues were detected using ELISA kits. Using H&E staining and immunofluorescence staining, morphological alterations and neuroinflammation in the hippocampus were assessed. To assess the impact of WDD on gut microbiota, 16S rDNA gene sequencing was done.

**Results:**

Mice in the CUMS group showed less food intake and less weight gain. Behavioral assessments revealed increased behaviors that resembled anxiety. WDD therapy reduced the mice’s anxiety-like behaviors while increasing their body weight and food intake. In addition, WDD treatment significantly enhanced gut microbiota diversity and effectively modulated composition. WDD also reduced pro-inflammatory cytokine levels in colonic and hippocampal tissues, alleviating intestinal inflammation and neuroinflammation.

**Conclusion:**

WDD ameliorates CUMS-induced anxiety by modulating gut microbiota and attenuating neuroinflammation in mice.

## 1 Introduction

Anxiety is an evolutionarily conserved adaptive response that serves to shield organisms from potentially life-threatening situations. However, when anxiety becomes chronic and dysregulated, it is classified as a pathological condition, profoundly impairing daily subjective experiences, behavior, and overall mental health. Anxiety is the most prevalent mental health disorder globally, affecting an estimated 25% of the population worldwide (Chellappa and Aeschbach, 2022). Accounting for 3.3% of the global burden of disease, anxiety disorders have become the ninth major cause of health-related disability (Penninx et al., 2021). Chronic stressful life experiences are among the environmental elements significantly influencing its etiology (Chang and Grace, 2014). Under unfavorable circumstances, such as persistent and uncontrollable stress, often triggered by social pressure or negative emotional stimuli, can cause anxiety disorders (da Silva et al., 2018). As a key component of memory and cognition, the hippocampus is one of the brain’s most sensitive and adaptable areas to stress stimulation. Memory loss and cognitive impairment are symptoms of anxiety, indicating that hippocampal dysfunction contributes to the pathogenesis of anxiety disorder (Pannekoek et al., 2015). Chronic stress affects hippocampus neurogenesis and causes neuroinflammation, contributing to emotional and cognitive deficits (Du Preez et al., 2021). The chronic unpredictable mild stress (CUMS) model, designed to mimic daily human stressors, induces behavioral and physiological changes in rodents resembling human anxiety and depression (Henningsen et al., 2012; Du Preez et al., 2021). CUMS exposure promotes passive coping strategies, increasing susceptibility to these disorders (Henningsen et al., 2012; Chang and Grace, 2014).

Gut microbiota, the largest and most direct external microenvironment, are essential to preserving human health and well-being (Hornig, 2013). They modulate energy metabolism, immune system development, and other critical physiological processes. The onset, progression, and remission of neuropsychiatric diseases are intimately related to alterations in gut microbiota. The “microbe-gut-brain axis” is fundamental for sustaining brain homeostasis and regulating normal behavioral patterns (Sharon et al., 2016; Morais et al., 2021). It is seen as a possible therapeutic target for stress behavioral impairments that can modulate both peripheral and brain immune landscapes (Westfall et al., 2021). The change in gut microbiota significantly impacts the pathophysiology of anxiety and depression disorders (Chen et al., 2018; Li et al., 2023). Numerous studies have demonstrated that gut ecology will become dysfunctional under chronic stresses, potentially resulting in systemic inflammation (Rudzki and Maes, 2020). Systemic inflammatory mediators can trigger the activation of neuroglial cells, which subsequently produce a cascade of inflammatory cytokines, amplifying the initial inflammatory response. These findings highlight gut microbiota as key players in anxiety/depression pathogenesis and potential targets for treatment (Slykerman et al., 2017).

Traditional Chinese medicine (TCM) offers multicomponent, multitarget therapeutic potential (Li et al., 2021). Wendan decoction (WDD) is a representative formula from *Ji Yan Fang* (Southern and Northern Dynasties), and adopted in the *Ancient Classical Chinese Medicine Formula Catalogue (First Edition)* (Wang et al., 2021). WDD is extensively utilized for managing neuropsychiatric, respiratory, and gastrointestinal disorders (Liu et al., 2009), with a particular emphasis on treating neurological and mental disorders, including schizophrenia, insomnia, and depression (Che et al., 2016; Wieland and Santesso, 2017). In clinical trials, WDD has shown significant efficacy in alleviating anxiety and depression symptoms, exhibiting favorable safety and therapeutic outcomes(Yang et al., 2023). Recently, a study has found potential WDD targets for the therapy of generalized anxiety disorder (GAD) using network pharmacology. It validated that WDD may inhibit the increase of anxiety behaviors and the proportion of interleukin-6 (IL-6) -positive lymphocytes in mice and reverse the PI3K/AKT signaling pathway and MAPK signaling pathway (Jin et al., 2022). However, it remains unclear whether WDD can alleviate stress-induced anxiety via gut microbiota modulation or neuroinflammation relief. Using CUMS model, this study investigates WDD’s behavioral, microbial, and neuroinflammatory effects in chronically stressed mice, providing some insights for future research.

## 2 Materials and methods

### 2.1 Animals and groups

Six-week-old male C57BL/6 J mice weighing 18–22 g were acquired from Beijing HFK Bio-Technology Co., Ltd., (Animal Production Licence No.: SCXK (Beijing) 2024–0003). The mice were acclimatized for 1 week in a specific pathogen-free (SPF) facility (ambient temperature maintained at 22 ± 1 °C, relative humidity at 55 ± 5%, under an automated light cycle of 12:12 h) with unlimited access to food and water. After acclimatization, mice were weighed and randomly divided into four distinct groups: (1) the control group (Con), (2) the CUMS model group (CUMS), (3) CUMS+diazepam (DZP) group (DZP), (4) CUMS+WDD group (WDD), with 10 mice per group, and each mouse housed in the separate cage. Over 28 days, the Con group received standard food and water, while mice in CUMS, DZP, and WDD groups were subjected to nine different CUMS stressors, following a protocol adapted from Wang et al. (2020). Detailed stressor schedules were provided in Supplementary material 1. Animal experiment procedures were approved by the Laboratory Animal Welfare and Ethics Committee of Henan University of Chinese Medicine (Approval No. IACUC-202410038).

### 2.2 Medicine

Consistent with previous studies (García-Ríos et al., 2019), DZP chosen as the positive control medication and administered daily via intragastric gavage at 2 mg/kg. WDD consists of six herbal ingredients (Henan Zhangzhongjing Pharmacy Co., Ltd., Henan, China), including *Pinellia ternata* (Thunb.) Makino (6 g), *Zingiber officinale* Roscoe (12 g), *Citrus aurantium* L. (9 g), Aurantii fructus Immaturus (6 g), *Bambusa tuldoides* Munro (6 g), Glycyrrhiza uralensis Fisch. ex DC. (3 g). Mice received 9.1-fold therapeutic doses of humans (body weight 60 kg) based on body surface area ratio (Jin et al., 2022), and the dose for mice was 6.37 g/kg/d (raw herbs). Detailed preparation processes of WDD are provided in Supplementary material 2.

### 2.3 Drug administration and sample collection

**Figure 1:**
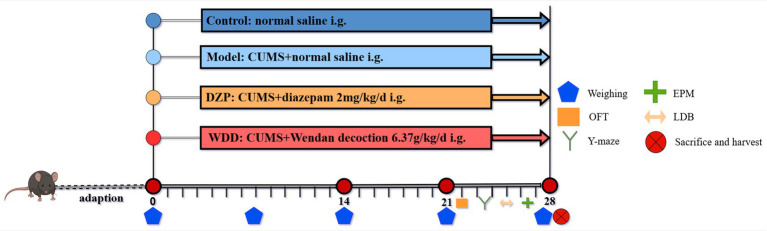
Experimental timeline and treatment schedule.

Mice in Con and CUMS groups received 0.1 mL/10 g normal saline, while intervention groups (DZP and WDD) were administered equivalent volumes of respective treatments. For 28 days in a row, treatments were given once daily. 1 h before stress exposure. Figure 1 shows the experimental timeline and treatment plan. Upon completion of experiment, samples were collected following the protocol detailed in Supplementary material 3.

### 2.4 Behavioral tests

The open field test (OFT), elevated plus-maze (EPM), and light/dark box (LDB) test were among the behavioral tests used to evaluate anxiety-like behaviors after medication administration. Spatial memory was evaluated via the Y-maze test. VisuTrack software (Shanghai Xinruan Information Technology Co. Ltd., Shanghai, China) was used to conduct and analyze all behavioral tests. All tests were conducted in a sequence of escalating stimulus intensity to minimize stress and habituation effects on mice. Detailed experimental procedures for behavioral tests were provided in Supplementary material 4.

### 2.5 Enzyme-linked immunosorbent assay (ELISA)

Hippocampal and colonic tissues from all mice were analyzed for interleukin-1β (IL-1β) (mlC50300B), IL-6 (ml098430B), and tumor necrosis factor-*α* (TNF-α) (mlC50536B) using ELISA kits (Shanghai Enzyme-linked Biotechnology Co., Ltd.). At 450 nanometers, the optical density (OD) value of each sample was measured using a microplate reader. ELISA Calc was used to plot a standard curve and calculate each sample’s concentration.

### 2.6 Hematoxylin and eosin (H&E) staining and immunofluorescence (IF) staining

H&E staining (Feldman and Wolfe, 2014) was used to assess morphological alterations of colonic and hippocampal tissues. Anti-Iba-1 mouse monoclonal antibody (GB15015, Servicebio, diluted 1:500) and anti-Arg-1 rabbit monoclonal antibody (GB11285, Servicebio, diluted 1:500) were used to perform IF staining (Zhang et al., 2023). Detailed protocols are provided in Supplementary material 5.

### 2.7 16S rDNA gene sequencing

16S rDNA gene sequencing was conducted by Majorbio Bio-pharm Technology Co., Ltd. (Shanghai, China). Fecal samples’ genomic DNA was extracted, and its integrity, concentration, and purity were verified. Subsequently, PCR amplification was performed using barcoded primer pairs targeting 16S rDNA gene. The resultant PCR amplicons were sequenced using an ABI GeneAmp® Model 9,700 PCR instrument. Sequences were categorized into Operational Taxonomic Units (OTUs) using Usearch 11 (version 11; accessible at^1^) for sequence analysis. A similarity criterion of ≥97% was applied. OUT taxonomic annotation of species was conducted to refer to the NT_16S (v20221012) using the RDP classifier (version 2.13; available at^2^), with a confidence threshold of 70%. The community composition of each sample was examined at several taxonomic levels. The PICRUSt2 (version 2.2.0) program and the R language (version 3.3.1) Tax4Fun (0.3.1) package were used to conduct functional prediction analysis.

### 2.8 Statistical analysis

Data are expressed as mean ± SD, and GraphPad Prism (v 9.5.0) was applied for statistical analyses and graph generation both. Data were analyzed by one-way analysis of variance (ANOVA) or Kruskal-Wallis test. The linear discriminant analysis (LDA) score threshold was set at more than 3.5. A *p*-value<0.05 or <0.01 was found to be statistically significant. Spearman correlation analysis (R v3.3.1, pheatmap package) was employed to assess the relationship between gut microbiota and inflammatory cytokines.

## 3 Results

### 3.1 WDD treatment increased body weight and food intake in CUMS-induced mice

**Figure 2:**
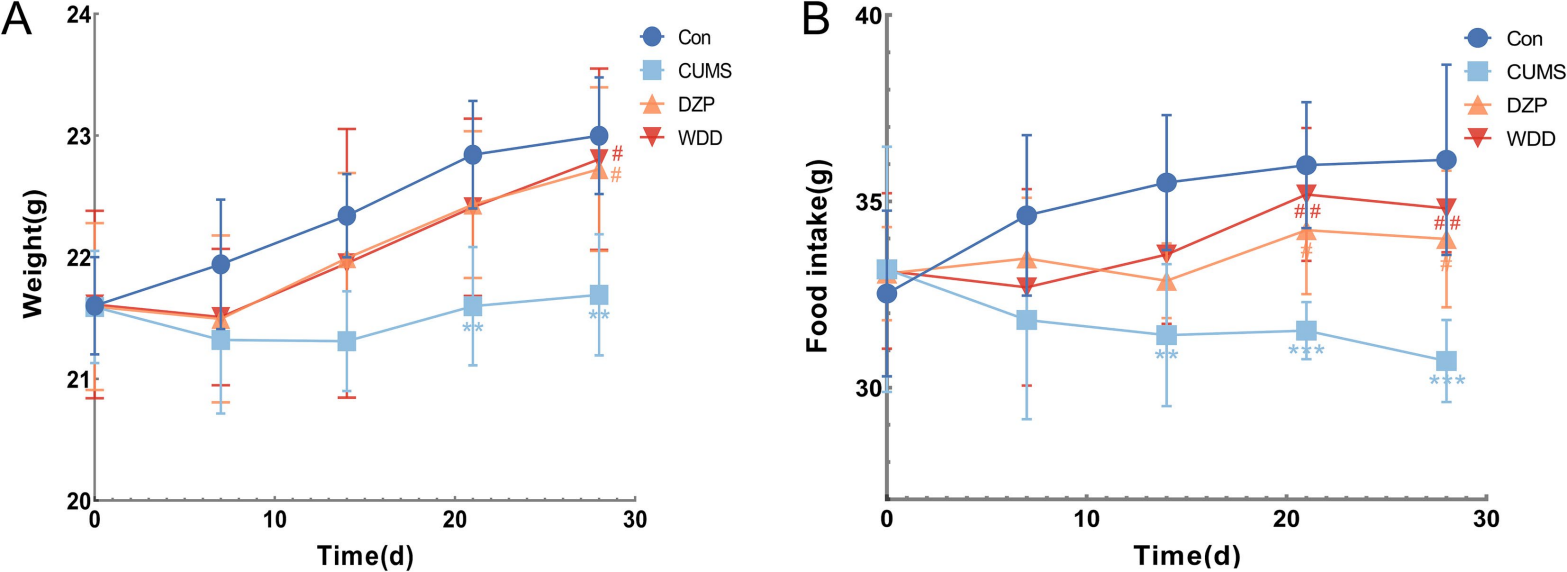
Effects of WDD on body weight and food intake in CUMS-induced mice. **(A)** Weekly body weight of mice in each group (*n* = 6). **(B)** Weekly food consumption of mice in each group (*n* = 6). ^**^p < 0.01, ^***^p < 0.001 vs. Con group; ^#^*p* < 0.05, ^##^*p* < 0.01 vs. CUMS model group.

Weekly body weight and food intake were recorded at corresponding times throughout the 4-week experiment (Figures 2A,B). Mice in the Con group exhibited steady weight gain. CUMS group showed significantly reduced weight gain compared to the Con group (*p* < 0.01). Both WDD and DZP treatments significantly improved weight gain relative to the CUMS group (*p* < 0.05). Food intake decreased significantly in the CUMS group (*p* < 0.01). WDD and DZP therapy significantly increased weekly food intake (*p* < 0.05).

### 3.2 WDD treatment attenuates anxiety-like behaviors in CUMS-induced mice

**Figure 3:**
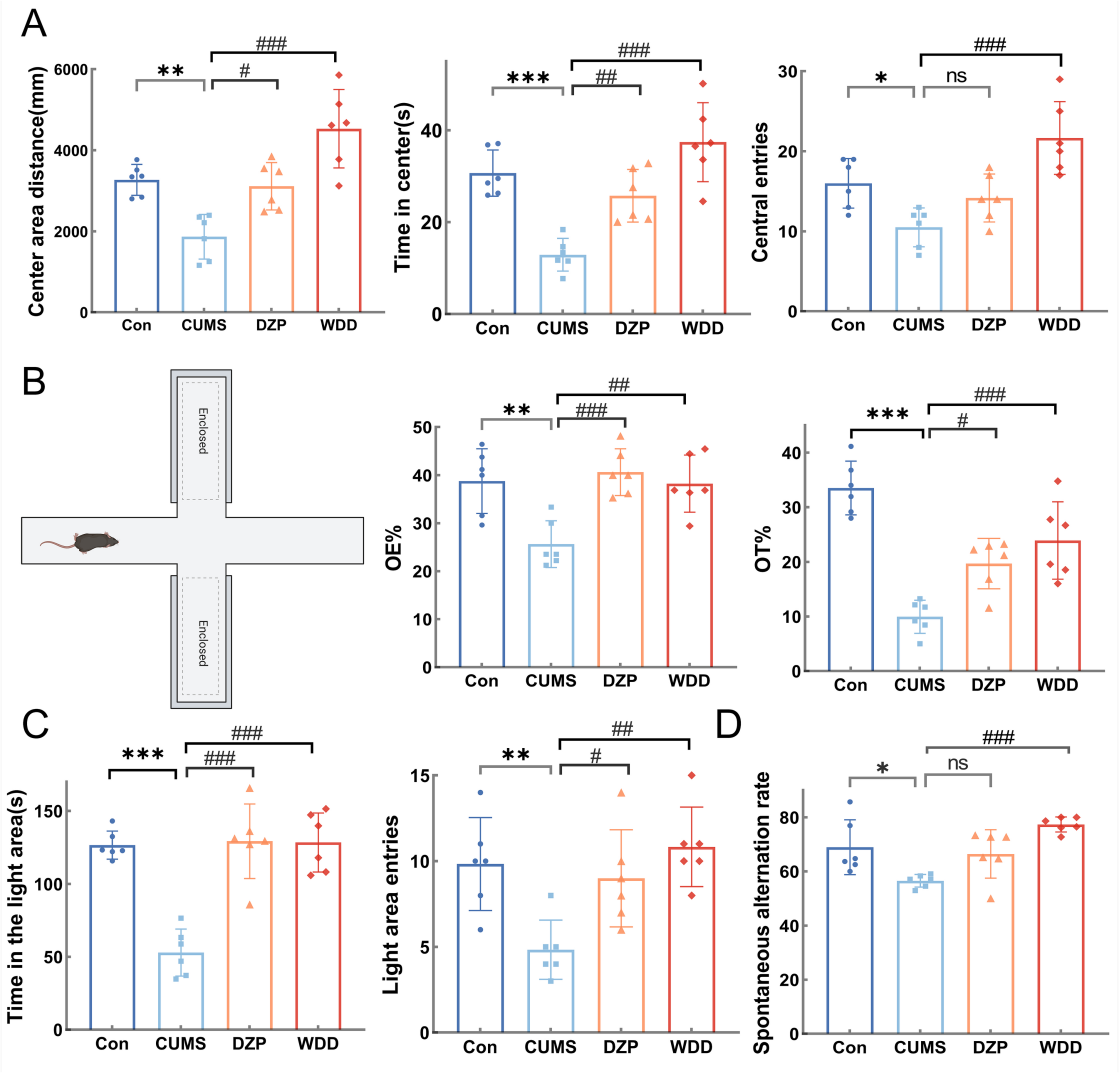
Effects of WDD on anxiety-like behaviors. **(A)** Results of the OFT experiment (*n* = 6). **(B)** Results of the EPM test (*n* = 6). **(C)** Results of the LDB test (*n* = 6). **(D)** Results of the Y-Maze test (*n* = 6). ^*^p < 0.05, ^**^p < 0.01, ^***^p < 0.001 vs. Con group; ^#^*p* < 0.05, ^##^*p* < 0.01, ^###^ *p* < 0.001 vs. CUMS model group; ns, not significant.

We assessed the ameliorative effects of WDD on behaviors resembling anxiety in mice produced by CUMS. In the OFT (Figure 3A), CUMS group exhibited reduced central area distance (*p* < 0.01), time spent in the center (*p* < 0.001), and the number of central entries (*p* < 0.05). Compared to the CUMS group, both WDD and DZP reversed these deficits (*p* < 0.05). In the EPM (Figure 3B), CUMS mice showed fewer open-arm entries (*p* < 0.01) and reduced time in open arms (*p* < 0.001). Both WDD and DZP intervention increased the time spent and percentage entries in open arms of mice (*p* < 0.05). In the LDB (Figure 3C), CUMS mice stayed longer in the dark environment (*p* < 0.001) and entered the light areas less frequently (*p* < 0.01). Following WDD treatment, the prolonged time of stay in the light area markedly increased (*p* < 0.001), along with a frequency of entries (*p* < 0.01). In the Y-maze test (Figure 3D), the CUMS group demonstrated a lower spontaneous alternation rate (*p* < 0.05), indicating impaired memory function due to CUMS exposure. WDD restored alternation rates to near-normal levels (*p* < 0.001).

### 3.3 WDD improves the morphology of hippocampal neurons in CUMS-induced anxious mice

**Figure 4:**
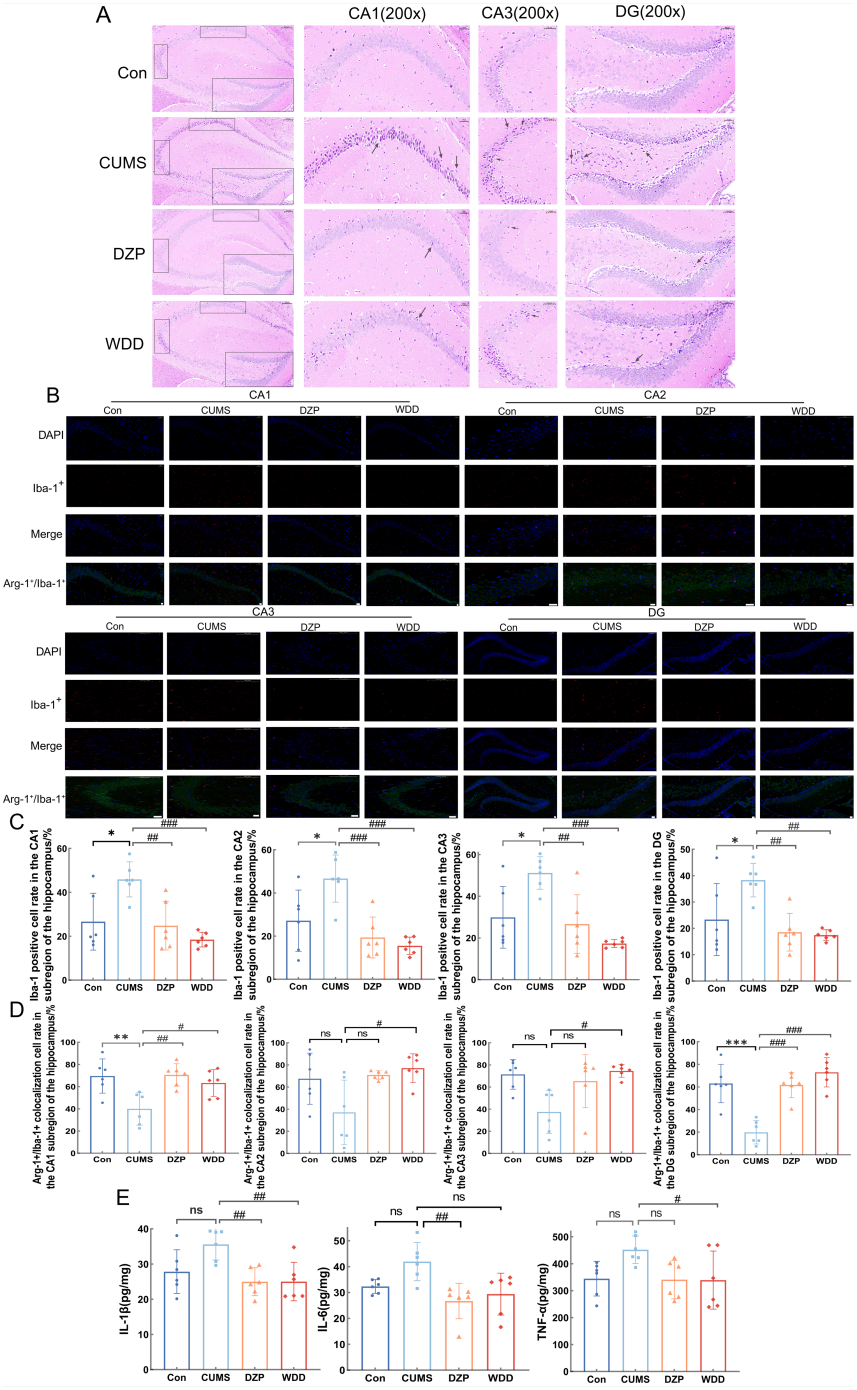
Effects of WDD on morphological alterations and neuroinflammation in the hippocampus. **(A)** Results of the hippocampal tissue H&E staining. CA1, CA3, and DG were chosen for comparison (*n* = 6). **(B)** Immunofluorescence staining results of hippocampus in Con, CUMS, DZP, and WDD groups. Iba-1^+^ cells are seen in red, Arg-1^+^ cells in green, and nuclei are stained with DAPI, resulting in a blue appearance. Scale bar = 100 μm. **(C)** Quantification of the Iba-1^+^ cell in the hippocampus (*n* = 6). **(D)** Quantification of the Arg-1^+^/Iba-1^+^ colocalization cells in the hippocampus (*n* = 6). **(E)** Hippocampal levels of pro-inflammatory cytokines (IL-1β, IL-6, and TNF-*α*) (*n* = 6). ^*^p < 0.05, ^**^p < 0.01, ^***^p < 0.001 vs. Con group; ^#^*p* < 0.05, ^##^*p* < 0.01, ^###^*p* < 0.001 vs. CUMS model group; ns, not significant.

H&E staining (Figure 4A) showed cells exhibited regular morphology in the Con group, with neurons arranged neatly and densely. Cells had tight intercellular junctions, intact nuclear membranes, clear nucleoli, abundant cytoplasm, and evenly distributed chromatin. In the CUMS group, cells showed a reduced number of layers, and neurons were disorganized. Intercellular arrangements were loose and irregular, exhibiting nuclear chromatin aggregation and deep staining. After WDD and DZP treatments, the number of cell layers increased in the hippocampus, and the neuronal organization disorder was restored, cells were more regularly arranged, and the chromatin was evenly distributed.

### 3.4 WDD mitigates neuroinflammation in CUMS-induced anxious mice

IF analysis examined Iba-1^+^ and Arg-1^+^/Iba-1^+^ cells in the hippocampus. The CUMS group had significantly more activated microglial cells in the four subregions of the hippocampus (CA1, CA2, CA3, and DG) than the Con group (*p* < 0.05). WDD therapy dramatically recovered the quantity of activated microglial cells in the hippocampus when compared to the CUMS group (*p* < 0.01) (Figures 4B,C). Furthermore, mice exposed to CUMS had significantly fewer Arg-1^+^ microglial cells in CA1 and DG than the Con group (*p* < 0.01). However, CA2 and CA3 showed no significant differences (*p* > 0.05). WDD treatment effectively reversed these changes (*p* < 0.05, Figure 4D). The hippocampal levels of pro-inflammatory cytokines were reduced by WDD therapy and were nearly normal (*p* < 0.05, Figure 4E). These findings collectively point to a conclusion that WDD’s anxiolytic and effects are linked to neuroinflammation modulation.

### 3.5 WDD alleviates colonic inflammation in CUMS-induced anxious mice

**Figure 5:**
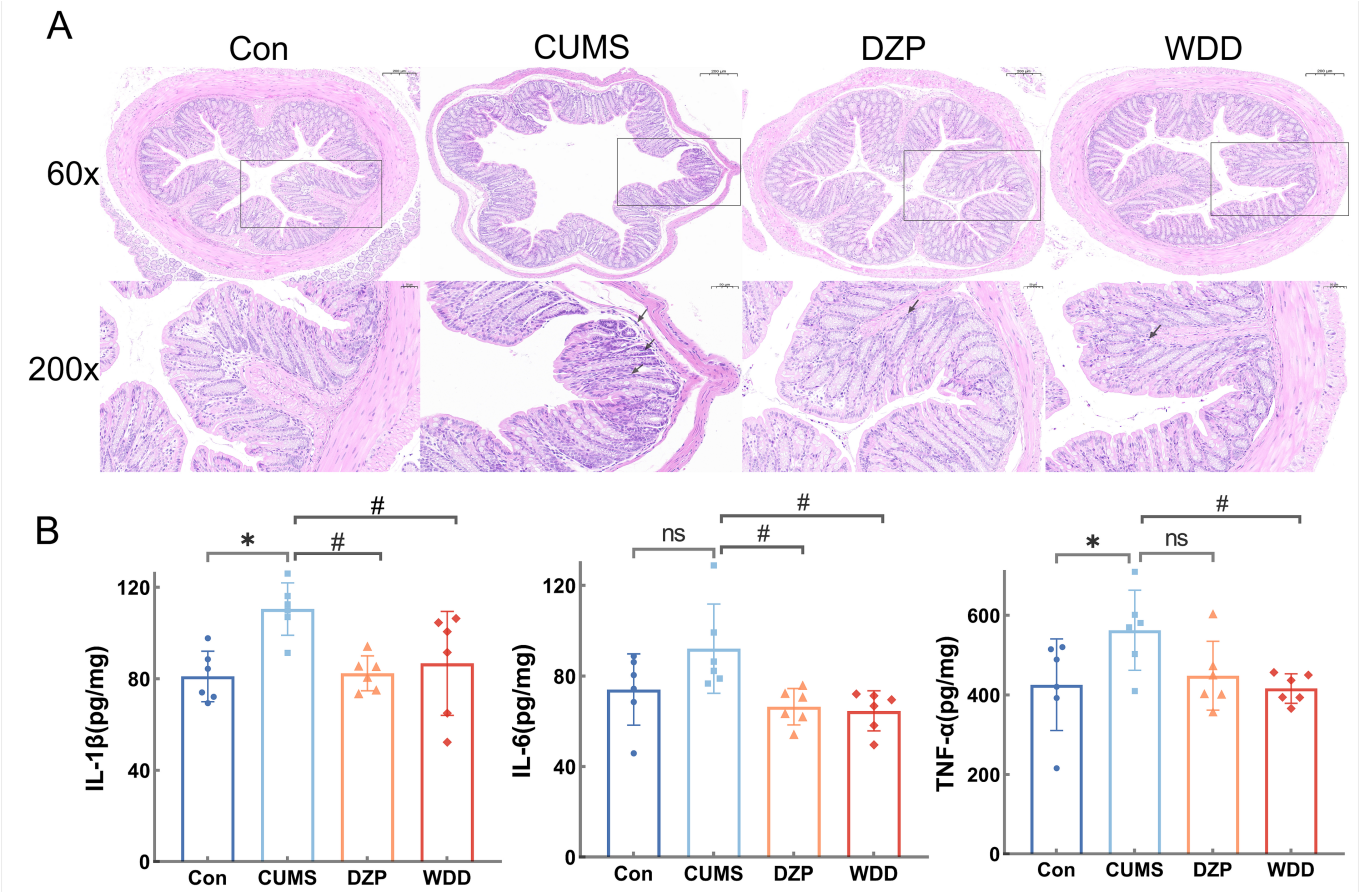
Effects of WDD on colonic inflammation. **(A)** H&E staining results of the effect of WDD on the colon pathology. **(B)** Effects of WDD on IL-1β, IL-6, and TNF-α levels of the colon (*n* = 6). ^*^p < 0.05 vs. Con group; ^#^*p* < 0.05 vs. CUMS model group; ns, not significant.

Histopathological study of the colonic tissue was performed using H&E staining (Figure 5A). The Con group had intact colonic mucosa, characterized by orderly epithelial cell arrangement and an absence of inflammatory cell infiltration. In the CUMS group, colonic mucosa exhibited substantial damage or absence, with the glands in lamina propria compromised, accompanied by considerable inflammatory cell infiltration. WDD treatment ameliorated mucosal damage and reduced inflammatory infiltration.

We assessed the impact of WDD on colonic inflammation. While there was no discernible difference in the levels of IL-6 expression (*p* > 0.05), CUMS mice significantly raised the expression levels of TNF-*α* and IL-1β in the colon (*p* < 0.05). WDD significantly reduced the expression of pro-inflammatory cytokines in comparison to the CUMS group (*p* < 0.05, Figure 5B), confirming its efficacy in mitigating CUMS-induced colonic inflammation.

### 3.6 WDD improves gut microbiota dysbiosis in CUMS-induced anxious mice

**Figure 6:**
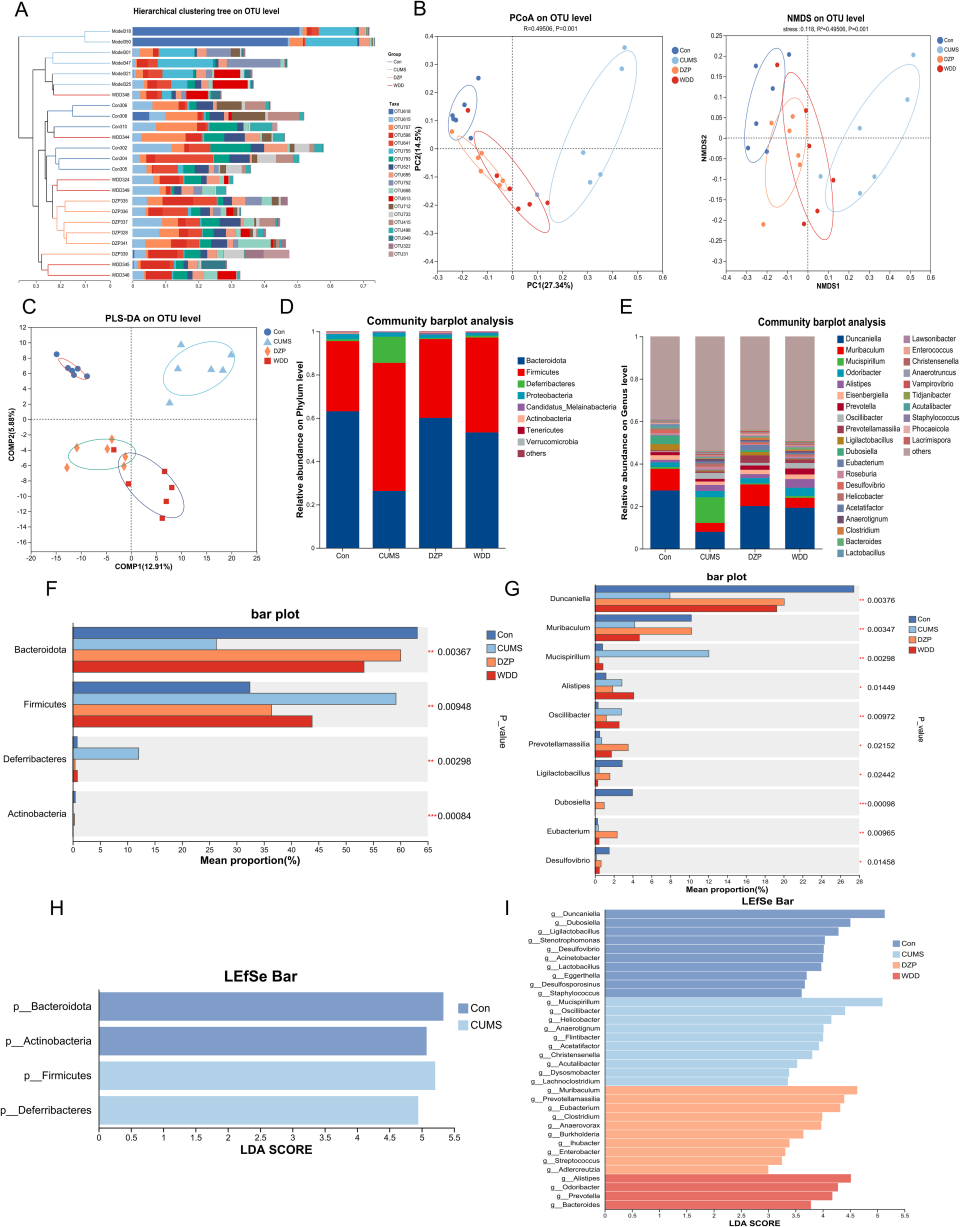
Effect of WDD on gut microbiota. **(A)** The hierarchical cluster analysis dendrogram (lengths between the branches represent distances between the samples). **(B)** Results of PCoA and NMDS for gut microbiota utilizing bray_curtis distance metrics (*n* = 6), stress<0.2, indicate that the graph has some interpretative significance. **(C)** Partial least squares discriminant analysis (PLS-DA) differences between groups. **(D)** Taxonomic profile in mice at the phylum level (*n* = 6). **(E)** Taxonomic profile in mice at the genus level (*n* = 6). **(F)** Notable variations in phylum-level species abundance. **(G)** Significant differences in species abundance at the genus level. **(H)** LEfSe analysis of Con and CUMS groups at the phylum level. **(I)** LEfSe analysis of all groups at the genus level. ^*^p < 0.05, ^**^p < 0.01, ^***^p < 0.001.

To assess whether WDD can modulate the gut microbiota of CUMS-induced anxious mice, 16S rDNA gene sequencing was performed across groups. Pan analysis (Supplementary Figure 1A) demonstrated sufficient samples and exhibited high microbiota species richness. Core analysis revealed that the number of microbiotas differed among groups (Supplementary Figure 1B). Rank-Abundance curves had responded to species abundance and species evenness (Supplementary Figure 1C). Chao, Shannon and Simpson indices showed no discernible differences in species richness between groups (*p* > 0.05, Supplementary Figures 1D–F). Hierarchical cluster analysis dendrograms uncovered substantial differences in gut microbiota abundance between the CUMS group and the Con group, with the WDD group exhibiting greater similarity to the Con group (Figure 6A). Principal component analysis (PCA) was used to conduct species diversity and characteristic species analysis and evaluate the similarity among microbial communities. PCoA, NMDS, and PLS-DA revealed an apparent separation of microbial communities in the CUMS group, with DZP and WDD groups clustering closer to the Con group (Figures 6B,C).

Subsequently, we looked into the microbiota’s relative abundance at various taxonomic levels. At the phylum level (Figure 6D), the CUMS model group exhibited a notable reduction in Bacteroidetes abundance (*p* < 0.001) and an increase in Firmicutes (*p* < 0.01) and Deferribacteres abundance (*p* < 0.001). These changes were reversed following WDD and DZP treatment. At the genus level (Figure 6E), in comparison to the Con group, the CUMS group showed a significant increase in the abundance of *Mucispirillum* (*p* < 0.001) and *Helicobacter* (*p* < 0.001) and a significant decrease in *Duncaniella* (*p* < 0.001) and *Muribaculum* (*p* < 0.05). WDD therapy reinstated the *Duncaniella* population (*p* < 0.05) and considerably reduced the populations of *Mucispirillum* (*p* < 0.001) and *Helicobacter* (*p* < 0.01) relative to the CUMS group. The Kruskal-Wallis H-test found 4 significantly different microbiota at the phylum level and 10 at the genus level (Figures 6F,G). LEfSe analysis identified key microbiota that differ in each group at the phylum/genus level. As depicted in Figure 6H, gut microbiota, including Bacteroidota, Actinobacteria, Firmicutes, and Deferribacteres played an important role in differentiating Con and CUMS model mice. Notably, *Muribaculum*, *Prevotellamassilia*, *Eubacterium*, *Clostridium*, *Anaerovorax*, and *Burkholderia* played an important role in the DZP-treated group, whereas the specific bacterial taxa in which the WDD intervention played a role were *Alistipes*, *Odoribacter*, *Prevotella*, and *Bacteroides* (Figure 6I). Investigating the functionality of gut microbiota in the treatment of WDD, we conducted an in-depth analysis of 16S rDNA gene profiles utilizing the PICRUSt2 program. Upon analyzing the COG database data (Supplementary Figure 1G), it was revealed that the microbiota function was predominantly associated with amino acid transport and metabolism, transcription, translation, ribosomal structure, and biogenesis. Furthermore, KEGG pathway analysis demonstrated that the predicted microbiota function was linked to carbohydrate metabolism, amino acid metabolism, membrane transport, and metabolism of cofactors and vitamins (Supplementary Figure 1H). These findings demonstrate WDD’s capacity to improve gut microbiota imbalance in CUMS-induced anxiety.

### 3.7 Association between gut microbiota and inflammatory cytokines in colonic/hippocampal tissues

**Figure 7:**
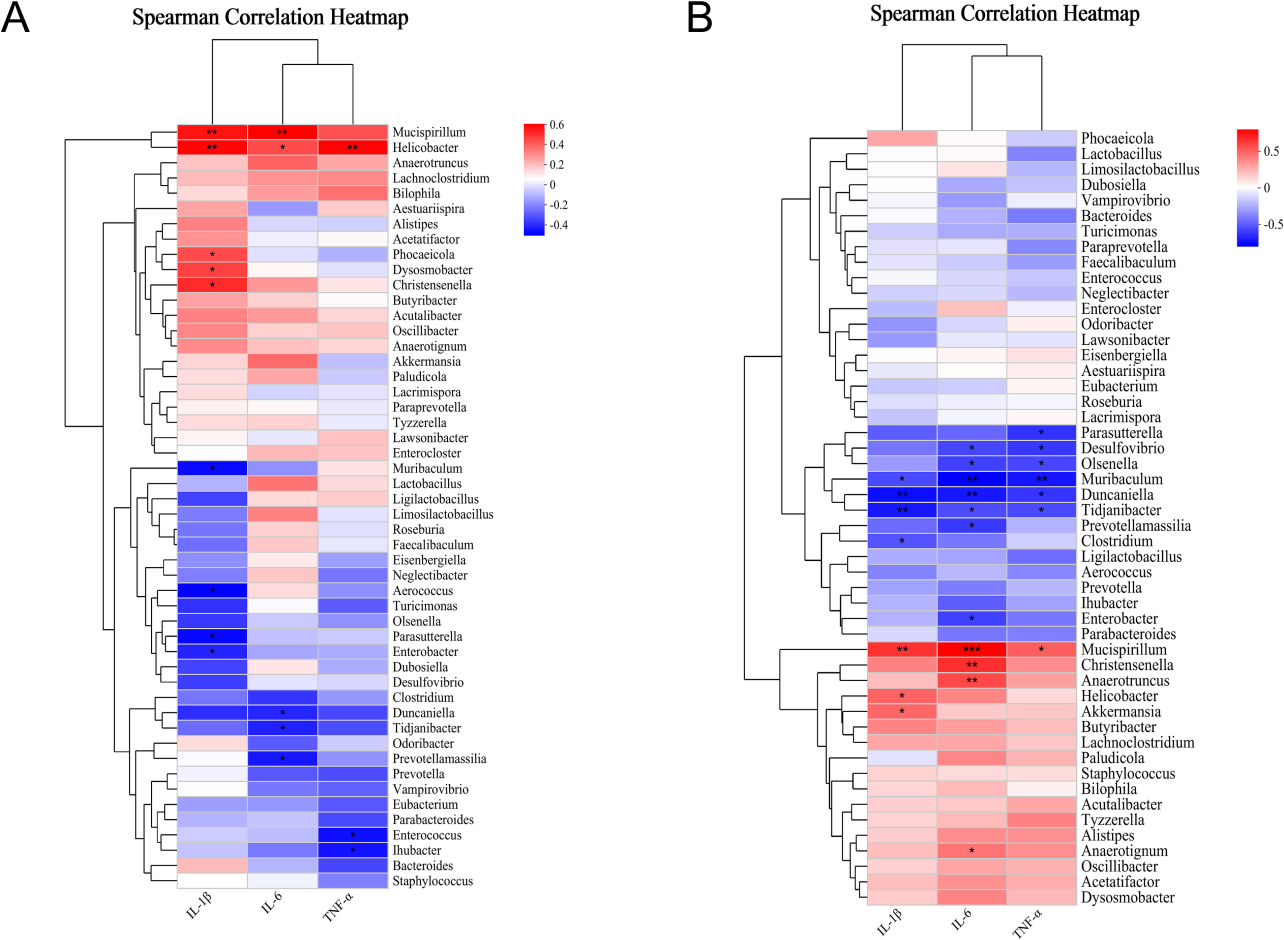
Relationships between gut microbiota and inflammatory cytokines in colonic/hippocampal tissues. **(A)** Correlation of gut microbiota (genus level) with pro-inflammatory cytokines in the colonic tissue (*n* = 6). **(B)** Correlation of gut microbiota (genus level) with pro-inflammatory cytokines in the hippocampal tissue (*n* = 6). ^*^p < 0.05, ^**^p < 0.01, ^***^p < 0.001.

Spearman correlation analysis revealed distinct relationships between gut microbiota and pro-inflammatory cytokines. Inflammation of the colonic tissue had a strong relationship with gut microbiota. *Mucispirillum* abundance correlated positively with IL-1β and IL-6 expression (*p* < 0.01). *Helicobacter* demonstrated a positive correlation with IL-1β, IL-6, and TNF-*α* expression (*p* < 0.05, Figure 7A). Inflammation of the hippocampus also had a strong relationship with gut microbiota. As shown in Figure 7B, the abundance of *Muribaculum*, *Duncaniella*, and *Tidjanibacter* was negatively correlated with IL-1β, IL-6, and TNF-α expression (*p* < 0.05), while *Mucispirillum* was correlated positively (*p* < 0.05). The abundance of *Christensenella* and *Anaerotruncus* exhibited a significant correlation with IL-6 expression (*p* < 0.01). *Helicobacter* and *Akkermansia* showed a positive correlation with IL-1β expression (*p* < 0.05). However, certain microbiota did not correlate with inflammatory markers, indicating that specific microbiota groups within the gut may serve distinct functions and not cause inflammation.

## 4 Discussion

Chronic or excessive stress can induce maladaptive responses and significantly disrupt the body’s equilibrium, producing several symptoms such as depression and anxiety (McEwen and Gianaros, 2011; Moritz et al., 2020). CUMS procedure has been proposed to replicate socio-environmental stressors to elicit behaviors associated with anxiety and depression (Guilloux et al., 2011). In this study, WDD demonstrated anxiolytic effects in CUMS-induced mice by modulating gut microbiota and attenuating neuroinflammation, aligning with our hypothesis and providing mechanistic insights into its therapeutic potential. CUMS mice exhibited significantly reduced weight gain and decreased food intake, consistent with previous reports (Li et al., 2019a; Wang et al., 2020). Following 28 days of CUMS exposure, CUMS mice demonstrated a range of behavioral alterations resembling anxiety, as verified by EPM, OFT, and LDB tests, validating model success. We observed that WDD treatment significantly restored weight gain and improved food intake, which may be related to mitigating intestinal inflammatory and injurious effects. Behavioral improvements included reduced anxiety-like behaviors in mice. The Y-maze test revealed WDD’s ability to restore spatial working memory deficits caused by chronic stress. Yang et al. (2012) demonstrated that enhancement of learning and memory in schizophrenic rats by WDD was equivalent to the impact of clozapine treatment.

Under normal conditions, the mammalian gut microbiota and the host maintain a state of microecological equilibrium, which is essential for overall health. Disruption of balance may promote the occurrence of diseases (Yoshikawa et al., 2013). It is frequently stated that the gut microbiota is often implicated in the neurobiology of social behavior and stress response (Dinan and Cryan, 2012; Cryan and Dinan, 2019). The composition of the gut microbiota is changed in a number of mental illnesses, such as anxiety and depression (Butler et al., 2023). 16S rRNA sequencing revealed that CUMS rats exhibited a significantly reduced concentration of Bacteroidetes and a significantly larger concentration of Firmicutes (Li et al., 2019b), suggesting microbial shifts contribute to stress pathology. Considering these factors, this study observed the regulatory effects of WDD on the gut microbiota of CUMS mice. As expected, examining the microbial makeup of mice feces indicated that WDD can mitigate gut dysbiosis. At the phylum and genus levels, we investigated the microbiota of each group of mice. The overall characterization of gut microbiota can be grasped at the phylum level, whereas the genus level allows for disease-specific association. In the CUMS model, at the phylum level, we discovered that while Bacteroidetes’ relative abundance declined, Firmicutes’ grew dramatically. It is consistent with Yue et al. (2021). Hydrolyzing proteins and carbohydrates is the primary job of Firmicutes in the stomach (Bäckhed et al., 2005). Bacteroidetes promote protein synthesis and the absorption of polysaccharides by primarily affecting steroids, polysaccharides, and bile acids (Xu et al., 2003). *Deferribacterium* is a conditionally pathogenic bacterium that promotes inflammation (Xie et al., 2023). *Deferribacterium* can generate energy through iron reduction, thereby playing a critical role in iron metabolism. We noted a substantial rise in the abundance of *Deferribacterium* in the CUMS group, which was markedly recovered following the WDD treatment. *Mucispirillum* is a pathogenic bacterium widely distributed in the gastrointestinal tract. Flagella help it to penetrate the mucosal barrier, potentially resulting in intestinal inflammation and colitis (Qin et al., 2012; Berry et al., 2015; Loy et al., 2017; Zhai et al., 2018). This study demonstrated that the abundance of *Mucispirillum* returned to normal after WDD treatment, indicating that WDD may mitigate intestinal inflammation by modulating gut microbiota composition. A cross-sectional study in humans reported that *Helicobacter pylori (Hp)* infection, the most common representative of *Helicobacter* spp., increases the risk of depression and mental disease. An elevated risk of psychological distress, anxiety, and depression was associated with higher Hp levels (Takeoka et al., 2017). Furthermore, research conducted by Hajime Suzuki et al.(Suzuki et al., 2019) demonstrated that *Hp* infection triggered anxiety and diminished food consumption among experimental animals, aligning with our observations. Marques et al. (2023) discovered that exposure to emotional single prolonged stress (E-SPS) elicited a phenotype resembling depression and anxiety, accompanied by a reduced relative abundance of *Duncaniella*. Our observations revealed that compared to the CUMS model, WDD treatment resulted in a notable reduction in the abundance of *Mucispirillum* and *Helicobacter*. Conversely, it brought about an increase in the abundance of *Duncaniella*. Through LEfSe analysis, we found that the most significantly different bacteria in WDD were *Alistipes*, which were proven beneficial gut microbiota (Zou et al., 2024). We conjectured that *Alistipes* are crucial in the functioning of WDD.

The gut-brain axis (GBA) is an attractive new target for psychiatric treatment. The emergence of stress-induced neuropsychiatric diseases is linked to GBA, including the regulation of inflammation, neurotransmitters, and their precursors (Butler et al., 2019; Butler et al., 2023). Research suggests that the gut, particularly the disordered microbiota, may be a major source of inflammation leading to neurodegeneration (Kesika et al., 2021; Buga et al., 2023). A previous study has reported that proteins associated with anxiety and depression in colitis accumulate within acute inflammatory response pathway (Yuan et al., 2021). One study used unbiased machine learning algorithms to formulate relevant GBA associations in the context of recurrent stress-induced anxiety and depression, proposing that the gut microbiota triggers the brain’s immune response and neuroinflammation (Westfall et al., 2021). Numerous studies have shown that changes to the gut microbiota impact inflammation and brain function. According to current studies, the gut can act as a conduit and send inflammatory signals to the brain through three different pathways: the neuronal pathway, the cellular immunological channel, and the systemic humoral pathway (Agirman et al., 2021). The permeability of body barriers, particularly the blood–brain barrier (BBB) and intestinal barrier, is compromised, allowing potentially harmful substances to enter the brain tissue along the GBA (Saffrey, 2013; Sarubbo et al., 2022). Intestinal toxins can get across the gut-blood barrier and into the systemic circulation when the intestinal barrier’s function is disrupted. These inflammatory mediators and toxins can therefore enter the brain through the blood–brain barrier. This disturbance sets off an inflammatory response that eventually results in neurological dysfunction, including anxiety, by activating immune cells like microglia in the brain (Parker et al., 2020). The body can also absorb gut bacterial metabolites such bile acids (Bhargava et al., 2020) and short-chain fatty acids (SCFAs) (Erny et al., 2015), some of which have protective effects on the blood–brain barrier by preventing microglial activation. In this study, we conducted a histological study of colon sections from each group and assessed the levels of IL-1β, IL-6, and TNF-*α* in the colon. According to the findings, CUMS activation exacerbated the colon’s inflammatory response, which is in line with what Chen et al. (2024). WDD treatment significantly ameliorated this change. Spearman correlation analysis showed that microbiota associated with intestinal inflammation were positively correlated with the levels of pro-inflammatory cytokines in the colon. In contrast, microbiota that improved inflammation were negatively correlated with the levels of pro-inflammatory cytokines in the colon (Huang et al., 2023). Similar results were shown in the hippocampal tissue. The limitation, however, is that we have not delved into the mechanisms that cause this correlation. Is intestinal permeability altered? Are the BBB and intestinal barriers disrupted? Do gut metabolites play a role in WDD treatment for anxiety? These will be the focus of our future research.

Neuroglia, resident immune cells in the brain, are highly responsive to various pathological stimuli, including stress, infection, and injury. Microglia are resident macrophages of the central nervous system (CNS) that are crucial for brain development, homeostasis, and disease (Wolf et al., 2017). Excessive activation and the resulting neuroinflammation can damage neurons and glial cells, even while microglial activation is essential for launching immune responses to pathogenic threats (Kocovski et al., 2021). Microglia engage with immune cells to initiate inflammation, culminating in the liberation of cytokines including TNF-α, IL-1β, IL-6, ROS, and nitric oxide (Lee et al., 2018), which can contribute to neuronal injury and cellular demise. We noted that stimulation with the CUMS program prompted the growth of hippocampus microglia and elevated the release of inflammatory cytokines. The link between microglial activation and behaviors resembling anxiety and depression is further supported by animal models (Riazi et al., 2015). WDD treatment reduced proinflammatory cytokines and increased Arg-1^+^ microglia. Arg-1^+^ microglia suppress inflammatory processes in the brain, promote neurite outgrowth, and protect neurons from inflammation-induced damage (Zhang et al., 2021; Jiang et al., 2022). While depression and anxiety are typically linked to elevated inflammation, evidence of “depression accompanied by immunosuppression” also exists that occurs in various people or at various phases of the illness. Patients with major depressive disorder have elevated levels of IL-6 and deficient maturation of NK cells and T helper cells, suggesting that both cellular immunosuppression and increased cytokine production can occur in the same individual. TNF is linked to atypical features and chronicity, and IL-6 may represent an “indicator of acute exacerbation status” (Beurel et al., 2020). Nevertheless, WDD is a complex concoction composed of numerous herbs, the precise actions and potential synergistic interactions among its constituents are yet to be elucidated. In the next phase of our study, we aim to identify the most important acting component in WDD and validate its efficacy through serum medicinal chemistry combined with network pharmacology. Moreover, there is still a lack of concrete proof that gut microbiota has a role in reducing anxiety through WDD. Consequently, future investigations will delve deeper into anxiolytic properties of WDD by employing fecal microbiota transplantation (FMT) and germ-free (GF) mice models, aiming to delineate the role of gut microbiota more clearly.

## 5 Conclusion

In conclusion, our research offers fresh perspectives on WDD from the standpoint of gut microbiota and neuroinflammation in reducing anxiety brought on by long-term, unmanageable stress. WDD likely exerts therapeutic effects by modulating gut microbial composition and attenuating neuroinflammatory responses. However, the mechanisms underlying WDD’s anxiolytic properties require further elucidation, particularly its multi-target pathways and gut-brain axis interactions, which warrant further investigation.

## Data Availability

The original contributions presented in the study are publicly available. This data can be found here: http://www.ncbi.nlm.nih.gov/bioproject/PRJNA1353653/.

## Footnotes

^1^ http://drive5.com/usearch/

^2^ http://rdp.cme.msu.edu/

## Glossary

CUMS: Chronic unpredictable mild stress
TCM: Traditional Chinese medicine
WDD: Wendan decoction
GAD: Generalized anxiety disorder
IL-6: Interleukin-6
SPF: Specific pathogen-free
DZP: Diazepam
OFT: Open field test
EPM: Elevated plus-maze
LDB: Light/dark box
ELISA: Enzyme-linked immunosorbent assay
IL-1β: Interleukin-1β
TNF-*α*: Tumor necrosis factor-α
OD: Optical density
H&E: Hematoxylin and eosin
IF: Immunofluorescence
OTUs: Operational taxonomic units
ANOVA: Analysis of variance
LDA: Linear discriminant analysis
PCA: Principal component analysis
Hp: *Helicobacter pylori*
E-SPS: Emotional single prolonged stress
GBA: Gut-brain axis
BBB: Blood–brain barrier
SCFAs: Short-chain fatty acids
CNS: Central nervous system
FMT: Fecal microbiota transplantation
GF: Germ-free
