# Supplementary material for: A new perspective on Wendan decoction: attenuation of CUMS-induced anxiety in mice by regulating gut microbiota and neuroinflammation
Source: Front Microbiol. 2025 Nov 21;16:1708868. doi: 10.3389/fmicb.2025.1708868 (PMC12679893; doi:10.3389/fmicb.2025.1708868)
Supplement: Supplementary file 1 [file Data_Sheet_1.docx]

Supplementary Material

# Supplementary Material 1

The chronic unpredictable mild stress (CUMS) protocol included nine randomized stressors: (1) 24 h of fasting; (2) 24 h of water fasting; (3) 12:12 h of light/dark cycle reversal (the light was switched on from 7:00 pm to 7:00 am and off from 7:00 am to 7:00 pm); (4) tail pinching (1 cm from tail tip, 3min); (5) cage tilt (45° for 24 h); (6) restraint stress (4 h); (7) swimming in cold water at 8°C (5 min); (8) swimming in hot water at 40°C (5 min); (9) cage crowding (2 h).

In order to prevent the animals from anticipating the stimuli, the aforementioned stimuli were placed randomly throughout one week, with two stimuli per day for four weeks. The same stimuli could not be presented in succession.

# Supplementary Material 2

The herbs were soaked in water for 1 h before decoction. Initially, 1000 mL of water was added, the mixture was brought to a boil over high heat, and then simmered it for 40 min to extract the herbal solution. 800 mL of water was added to the herbal residue and simmered for another 30 min. The two decoctions were filtered through gauze and concentrated using a rotary evaporator. The final concentration of the decoction was mixed and concentrated to 66 mL.

# Supplementary Material 3

After the experimental period, mice were fasted overnight and euthanized after 1 h the last dose. Anesthesia was induced via intraperitoneal injection of 0.1 mL/20 g of 1% sodium pentobarbital. Blood was collected into centrifuge tubes, clotted at room temperature for 30 min, and centrifuged at 3000 ×g for 15 min to isolate serum, which was stored at −80°C. Mice were perfused with saline solution, and the brain was rapidly dissected on ice. Half of the cerebral tissue was fixed in 4% paraformaldehyde protected from light (48 h, room temperature), followed by histopathological analysis, while the remaining half was dissected to isolate the hippocampus and cortex and stored at -80°C for biochemical analysis. The distal 1 cm segment of the colon was fixed in 4% paraformaldehyde; remaining tissue was stored at -80°C.

# Supplementary Material 4

Before all behavioral tests, mice were habituated in the testing room for at least 2 h.

The open field test (OFT) apparatus consisted of a white cube space (50 cm × 50 cm × 45 cm) with a camera above. The floor of the box was evenly divided into 25 zones, with 9 central zones and 16 surrounding zones. Mice were placed in the center of the space and allowed 5 min of free exploration; the system automatically recorded the central area distance, duration time, and number of central region crossings.

The elevated plus maze (EPM) test apparatus consisted of two oppositely positioned open arms (30 cm × 5 cm), two oppositely positioned closed arms (30 cm × 5 cm × 15 cm), and a central platform (5 cm × 5 cm), elevated 60 cm above the floor. Mice were placed in the center of the platform facing toward one of the open arms and allowed 5 min of free exploration. The number of entries and time spent in the open and closed arms were recorded.

The light/dark box (LDB) test apparatus comprised two compartments separated by a connecting door. The light compartment (24 cm × 20 cm × 20 cm) was illuminated by fluorescent lamps, while the dark compartment (12 cm × 20 cm × 20 cm) was completely devoid of light. Each mouse was placed in the light compartment facing the door and observed for 5 min. A video motion tracking system recorded the number of transfers between the two compartments and the time spent in the light compartment.

The Y-maze test was employed to evaluate spatial recognition memory in mice. The apparatus consisted of three opaque plastic arms labeled Ⅰ, Ⅱ, and Ⅲ, arranged at 120° angles relative to each other. Mice were placed at the intersection of the three arms each time. The order of entry into each arm was recorded over 5 min, where complete entry of a mouse's limb was defined as entry into that arm. The order of arm entries was analyzed, and the rate of spontaneous alternations was calculated using the formula: spontaneous alternation rate = [(number of spontaneous alternations) / (total number of arm entries - 2)] × 100%.

# Supplementary Material 5

Brain hemispheres and colon tissues were collected, fixed in 4% paraformaldehyde, and embedded in paraffin. Then, hematoxylin and eosin (H&E) staining was initiated. Tissue sections (5 µm thick) were prepared using a microtome. Sections were dewaxed in xylene and rehydrated through a graded ethanol series and stained with hematoxylin (5 min). After rinsing with tap water, the sections were differentiated in 1% HCl-alcohol for a few seconds, blued in 0.6% ammonium hydroxide and a final rinse with water. Sections were counterstained with eosin solution (2 min) and rinsed with running water. Sections were then dehydrated through a series of graded alcohols, cleared in xylene, and mounted with neutral resin.

Immunofluorescence (IF) staining: Paraffin-embedded sections underwent dewaxing, rehydration, and antigen retrieval (citrate buffer). Sections were blocked with 3% bovine serum albumin (BSA) (30 min, room temperature). Sections were incubated overnight at 4°C with primary antibodies, followed by secondary antibody incubation at room temperature away from light (50 min). After counterstaining with DAPI, the sections were sealed and observed under a fluorescence microscope. Double staining results in CA1, CA2, CA3, and DG subregions of the hippocampus were analyzed using Aipathwell® software (Wuhan servicebio technology Co., LTD).

# Supplementary Figures and Tables

## Supplementary Figure 1


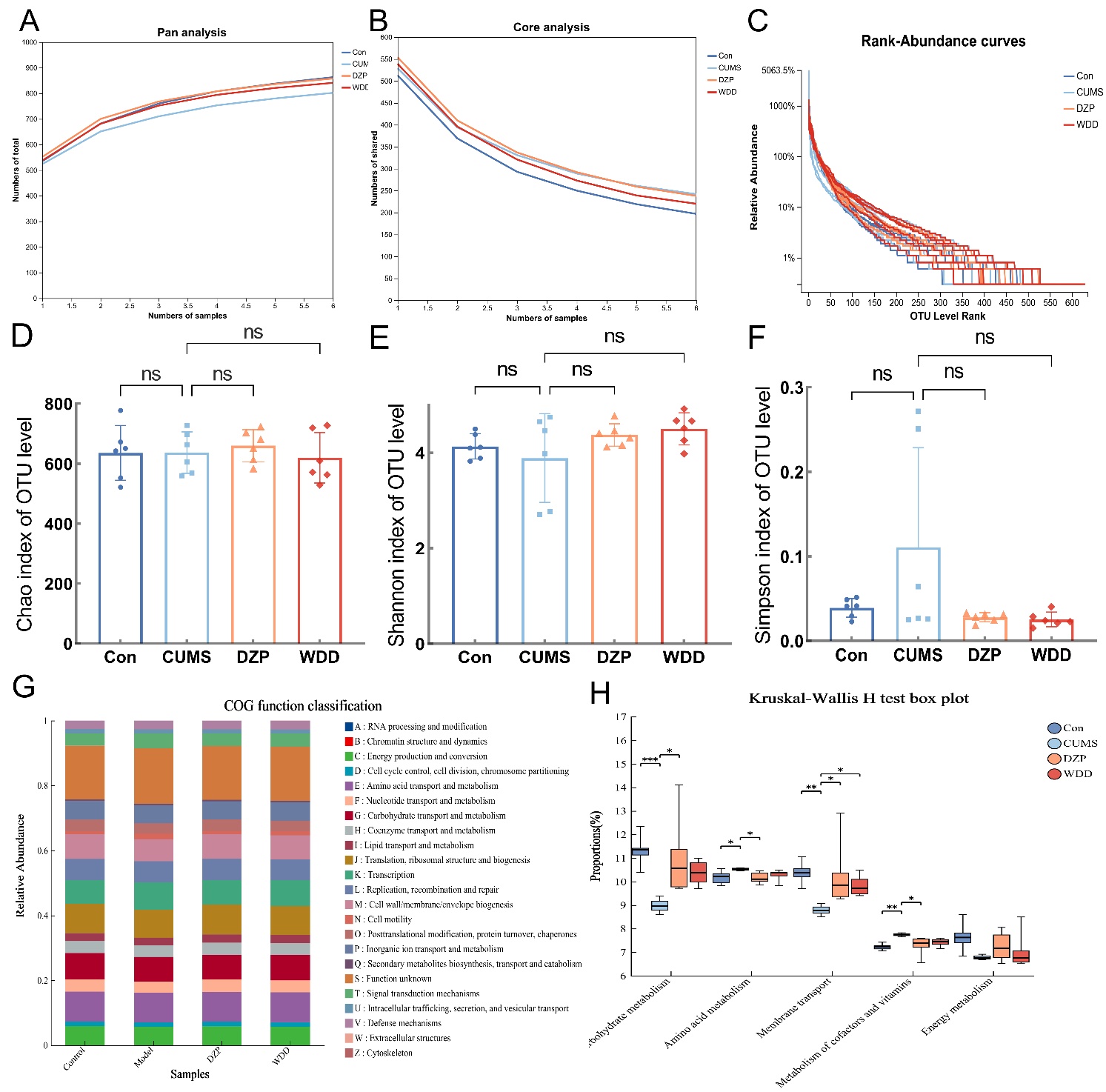


**Supplementary Figure.** Effect of WDD on gut microbiota. (A) Pan analysis. (B) Core analysis. (C) Rank-abundance curves. (D) Chao index. (E) Shannon index. (F) Simpson index. (G) PICRUST2 software for COG functional classification statistical bar charts. (H) Box plot of KEGG function prediction by Tax4Fun. ^*^ p < 0.05, ^**^ p < 0.01, ^***^ p < 0.001; ns, not significant.
